# Current Concepts of Biliary Atresia and Matrix Metalloproteinase-7: A Review of Literature

**DOI:** 10.3389/fmed.2020.617261

**Published:** 2020-12-21

**Authors:** Mark Nomden, Leonie Beljaars, Henkjan J. Verkade, Jan B. F. Hulscher, Peter Olinga

**Affiliations:** ^1^Divison of Pediatric Surgery, Department of Surgery, University of Groningen, Groningen, Netherlands; ^2^Division of Pharmaceutical Technology and Biopharmacy, Groningen Research Institute of Pharmacy, University of Groningen, Groningen, Netherlands; ^3^Division of Pediatric Gastroenterology and Hepatology, Department of Pediatrics, University Medical Center Groningen, University of Groningen, Groningen, Netherlands

**Keywords:** biliary atesia, progressive liver fibrosis, cholangiopathy, biliary fibrosis, Matrix metalloproteinase-7 (MMP)-7

## Abstract

Biliary atresia (BA) is a rare cholangiopathy of infancy in which the bile ducts obliterate, leading to profound cholestasis and liver fibrosis. BA is hypothesized to be caused by a viral insult that leads to over-activation of the immune system. Patients with BA are surgically treated with a Kasai portoenterostomy (KPE), which aims to restore bile flow from the liver to the intestines. After KPE, progressive liver fibrosis is often observed in BA patients, even despite surgical success and clearance of their jaundice. The innate immune response is involved during the initial damage to the cholangiocytes and further differentiation of the adaptive immune response into a T-helper 1 cell (Th1) response. Multiple studies have shown that there is continuing elevation of involved cytokines that can lead to the progressive liver fibrosis. However, the mechanism by which the progressive injury occurs is not fully elucidated. Recently, matrix metalloproteinase-7 (MMP-7) has been investigated to be used as a biomarker to diagnose BA. MMPs are involved in extracellular matrix (ECM) turnover, but also have non-ECM related functions. The role of MMP-7 and other MMPs in liver fibrosis is just starting to be elucidated. Multiple studies have shown that serum MMP-7 measurements are able to accurately diagnose BA in a cohort of cholestatic patients while hepatic MMP-7 expression correlated with BA-related liver fibrosis. While the mechanism by which MMP-7 can be involved in the pathophysiology of BA is unclear, MMP-7 has been investigated in other fibrotic pathologies such as renal and idiopathic pulmonary fibrosis. MMP-7 is involved in Wnt/β-catenin signaling, reducing cell-to-cell contact by shedding of E-cadherin, amplifying inflammation and fibrosis via osteopontin (OPN) and TNF-α while it also appears to play a role in induction of angiogenesis This review aims to describe the current understandings of the pathophysiology of BA. Subsequently, we describe how MMP-7 is involved in other pathologies, such as renal and pulmonary fibrosis. Then, we propose how MMP-7 can potentially be involved in BA. By doing this, we aim to describe the putative role of MMP-7 as a prognostic biomarker in BA and to provide possible new therapeutic and research targets that can be investigated in the future.

## Introduction

### Biliary Atresia

Biliary atresia (BA) is a rare cholangiopathy of infancy leading to obliteration of the intra- and extrahepatic bile ducts (1). The incidence of BA varies around the world from 1 case per 19,000 live births in Europe to 1 per 8,000 live births in eastern Asia (2–4). Infants that are affected present with conjugated hyperbilirubinemia, acholic stools, and dark urine (5). BA exists in an isolated or non-syndromic (IBA) form and a syndromic (SBA) form (6). The cause of both subtypes is currently unknown. It is thought, however, that SBA is caused by an error in development because other abnormalities of development are associated with this type, such as polysplenia, malrotation of the intestine and a pre-duodenal portal vein. The combination of BA and splenic malformation is a specific diagnostic subgroup, called Biliary Atresia Splenic Malformation variant (BASM) (7). Furthermore, BASM is characterized by mutations of the polycystic kidney disease 1 like 1 (*PKD1L*) gene that is associated with the rotation of the organs during embryonic development, supporting a developmental origin (8). IBA, on the other hand is thought to be caused by an (infectious) insult occurring somewhere between conception and the perinatal period (9, 10). IBA is characterized by a progressive inflammatory response resulting in injury to the bile ducts (9). The innate and adaptive immune system are therefore believed to play a prominent role in the pathophysiology of IBA. The innate immune system is the first line of defense of the immune system against pathogenic intruders. The adaptive immune system, on the other hand, is a defense system that is able to develop a very specific immune response against a pathogenic intruder (11, 12). Although there is a clear distinction, the two systems work hand in hand to rid the body of pathogens (11, 12).

Clinical diagnosis of BA is difficult; the golden standard for diagnosing BA therefore is an invasive liver biopsy or an endoscopic retrograde cholangio-pancreatography (ERCP) (1). The primary treatment of BA consists of the Kasai portoenterostomy (KPE) where bile flow is restored by removing the entire atretic extrahepatic bile duct and replacing it with a Roux-en-Y-loop of the intestine, so that bile can drain to the intestine (1, 5). KPE is deemed surgical successful when there is a potent connection between liver and intestine, allowing drainage. Therapeutic success of KPE treatment is evaluated according to the levels of bilirubin at 6 months after KPE. If clearance of hyperbilirubinemia is achieved (<20 μmol/L), KPE is deemed therapeutically successful. However, despite receiving a surgically and therapeutically successful KPE, liver fibrosis in BA patients often progresses to cirrhosis for which a liver transplantation (LTx) is required, making, BA is the most common indication for pediatric LTx (13).

Clearance of hyperbilirubinemia is associated with longer native liver survival (NLS) (14, 15). Patients that are treated at a younger age have a higher chance of achieving clearance of jaundice (3, 14). This may be related to the extent and amount of liver fibrosis that is present when KPE is performed. However, consistent proof for a relation between liver fibrosis and NLS is lacking. A relation between the aspartate transaminase-to-platelet ratio (a marker for liver fibrosis i.e.,) was not found to be correlated to NLS in BA patients, for example (16). Unfortunately, age at KPE is, as of yet, the only factor that can be used to estimate the prognosis of BA patients. Moreover, age at KPE is not a waterproof prognostic factor since most patients with a successful KPE still require an LTx eventually due to progressive liver fibrosis. This indicates that there may be other mechanisms than fibrosis due to a high level of bile acids/cholestasis involved in the progression of liver fibrosis. Immune mediated mechanisms of liver fibrosis, such as observed in related cholestatic conditions such as primary biliary cirrhosis (PBC) and primary sclerosing cholangitis (PSC), show similarities but are not the same as that observed in BA (17, 18). BA-associated liver fibrosis is a unique process with unique components playing a role in the pathogenesis that can be used for diagnosis and prognosis estimation. Matrix metalloproteinase-7 (MMP-7) has recently been investigated as a possible less invasive diagnostic marker in BA as alternative to an invasive liver biopsy or ERCP and shows good sensitivity and specificity (19–21).

### Matrix Metalloproteinases

MMP-7 belongs to the group of matrix metalloproteinase family (MMPs). During the initial discovery, MMPs were thought to fulfill a role in the remodeling of the extracellular matrix (ECM). As a family, MMPs are able to proteolytically cleave all the components of the ECM (e.g., collagens and fibronectin). MMPs can be produced and secreted by a variety of cells, ranging from connective tissue cells to macrophages (22). MMPs are classified in different groups according to their substrate and can act as pro- or antifibrotic proteases (23). The activity of the group of MMPs is controlled by tissue inhibitors of metalloproteinases (TIMPs) (24). In fibrotic pathologies there is an excess accumulation of connective tissue elements, such as collagen (25). Because of their involvement in remodeling of the ECM, MMPs have been investigated in fibrotic pathologies such as lung and liver fibrosis (26, 27).

Besides their function as remodelers of the ECM, numerous non-ECM related functions of multiple MMPs are being discovered, such as functions in inflammation (28, 29). MMPs are able to proteolytically cleave non-ECM proteins by a process termed “shedding.” For example, MMPs are able to activate cytokines, enhancing inflammation (30). Moreover, in cancer MMPs can enhance angiogenesis, while they can also reduce cell-to-cell integrity promoting tumor growth and invasion, respectively (31). For this reason, the role of various MMPs has been investigated in numerous pathologies, such as fibrosis and cancer in various organs (24).

### MMP-7

Studies have shown that MMP-7 is present in different quantities in the livers of BA patients with different postoperative outcomes, which provides circumstantial evidence that MMP-7 may play a role in the pathophysiology of BA (32, 33). While the potential of MMP-7 to diagnose BA is clear, there is not much research performed on where exactly in the pathophysiology of BA MMP-7 can be a factor. Moreover, MMP-7 expression correlates to the extent of liver fibrosis in BA patients at time of diagnosis. However, as liver fibrosis is an abnormal process of tissue regeneration, MMP-7 might merely be a (specific) marker of this abnormal regeneration and not play an active role in the pathogenesis of BA. However, as MMP-7 is suspected to be involved in BA, its exact role and significance in the progression of BA-related liver fibrosis requires further investigation.

This review aims to describe the current concepts of the, as of yet, elusive pathophysiology of BA. Then, we aim to describe the characteristics and functions of MMP-7. Subsequently, we aim to describe where in the pathophysiology of BA MMP-7 may be involved. By doing this, we aim to further define the relation between MMP-7 and BA thereby opening up new areas that can be targeted by future research.

## Etiology of Biliary Atresia

The etiology of BA is currently unknown. Much research has focused on the pathophysiology of IBA and this review will therefore discuss this subtype of BA.

One of the most popular hypotheses for the etiology of IBA is an infectious (viral) insult that leads to over activation of the immune system. The immune system subsequently targets the bile ducts and this leads to obliteration of the bile ducts (1, 9). This hypothesis has gained support due to the fact that histological evidence of inflammation in the liver and bile duct remnants at time of diagnosis in BA patients has been observed. Bill et al. (34) were the first of many studies to observe lymphocytes and other immune cells in biliary ducts of BA patients. Moreover, various viruses have been investigated in BA patients (35, 36).

Rhesus Rotavirus (RRV) has been used most often to create animal models that mimic BA and is considered the gold standard. Riepenhoff-Talty et al. (37) were the first that demonstrated the use of RRV type A in mice to mimic BA. The infected mice developed cholestasis and portal hepatitis within days after inoculation with RRV type A. A gene segment encoding a structural protein of RRV, VP4, was later identified as responsible for the specific activation of the murine immune system (38). VP4 specific inoculation of mice leads to a cholangiopathy mimicking BA, illustrating continuous improvement of the disease model (39–41).

Subsequently, studies have searched for serological and hepatic evidence of infection with Rotavirus, Reovirus or Cytomegalovirus (CMV) in BA patients (35, 36, 42). These studies showed mixed results: no conclusive evidence of a difference in infection rate of Rotavirus between BA patients and controls was observed. Studies that investigated Reovirus in infants with BA suffered from small sample sizes, lack of control groups in some studies and mixed results as well (42–44). However, recently CMV+ BA has been identified as a subgroup of BA, with a poorer prognosis than IBA patients (45). Mixed results can be due to a variety of reasons, such as an immature immune system of the BA infant resulting in a lack of proof or absence of infection or that there are multiple viruses that play a role in the activation of the immune system, for example (4, 46). Moreover, overactivation of the immune system that would eventually lead to BA via a virus can occur by mechanisms such as molecular mimicry or bystander activation. It is very hard to pinpoint to the exact virus or part of the viral genome that can cause the overactivation. It is possible that multiple viruses share a part of the genome, making it difficult to find consistent proof of infection with a single total virus in the livers of BA patients.

Other models of BA were recently described. The isoflavonoid biliatresone has been shown to selectively destruct the extrahepatic cholangiocytes in zebrafish (47). Biliatresone also mimicked BA in mice and was studied in cell cultures (48–50). These studies also investigated the mechanism of action by which the toxin could accomplish toxicity to the cholangiocytes. Biliatresone depletes glutathione (GSH), which causes disruption of microtubules in cholangiocytes (49). Microtubules are essential for lumen formation and polarity of epithelial cells and disruption of the tubules results in increased epithelial permeability and reduced cell-to-cell contact (49). RNA sequencing revealed downregulation of cell adhesion related genes (48). Therefore, Yang et al. speculate that a yet unidentified toxin may cause BA in humans by depletion of GSH (48).

The time at which the hypothesized insult leading to IBA occurs (prenatal or perinatal), remains to be determined. IBA is classically considered to originate in healthy new-borns with the insult occurring somewhere in the perinatal period with symptoms only appearing after the first weeks of life (1). Recently, evidence for BA as a disease starting *in utero*, such as hyperbilirubinemia within 24 h of life and abnormal gamma-glutamyl transferase (GGT) levels in the amniotic fluid of BA patients, is accumulating (51–53).

No conclusive evidence of what exactly causes IBA and when it is caused is available yet. There are various potential triggers that can lead to the same disease phenotype. Therefore, the cause of IBA can be multifactorial. However, regardless of the cause, the subsequent histological characteristics of immune mediated damage is similar among IBA patients. This will be described in the next section.

## Cell Mediated Immune Response in Biliary Atresia

### Innate Immune Response

Cholangiocytes are the biliary epithelial cells that line the extra and intrahepatic bile ducts. Cholangiocytes are heterogeneous and reactive types of cells (17). They are able to respond to a variety of foreign substances via pattern recognition receptors (PRRs). PRRs recognize Pathogen Associated Molecular Patterns (PAMPs) that are associated with specific types of microbes, so that a specific response against that microbe is elicited (54). Toll-like receptors (TLRs) are the most commonly described PRRs in humans. Monocytes, hepatic macrophages (i.e., Kupffer cells), dendritic cells (DCs) and cholangiocytes express various TLRs on their membrane (55). Each TLR has different specific ligands. TLR3 and TLR7 are activated by double stranded (ds) and single stranded (ss) viral RNA, respectively (56). Activation of TLRs induce the production of pro-inflammatory cytokines, chemokines, and other pro-inflammatory proteins allowing cells that express TLRs to actively participate in the immune response (12). The end result is the activation of the innate immune response that is required to clear the microbe that has infiltrated the tissue. It is likely that cholangiocytes play an active role in the pathogenesis of BA, but immune cells, such as Kupffer cells, probably play a more central role in producing and maintaining the immune response observed in BA (9, 57).

In BA, TLR activation has been investigated in liver tissue. Huang et al. (58) found increased hepatic expression of TLR7 in BA compared to choledochal cyst (CC) patients, another cholangiopathy with embryological origin (59). The increased expression was mainly located in the resident Kupffer cells and cholangiocytes in BA patients. Interestingly, they found a decreased hepatic expression of TLR7 in the late stage of BA compared to the early stage of BA, indicating that TLR7 may be more important in the initiation of the immune response rather its maintenance. Harrada et al. (60) found overexpression of TLR3 in liver tissue of BA patients compared to controls with a cholestatic liver disease. Both TLR3 and 7 induce an inflammatory cascade by inducing a type 1 interferon (IFN) response, regardless on which cell type the receptor is located as a response to viral RNA (12). Type 1 IFN uses human myxovirus resistance protein 1 (MxA) as a signaling molecule. MxA is a very specific marker of type 1 IFN signaling and was up-regulated in livers of BA patients (61). Up-regulation of TLR 3 and 7 in BA supports the finding of the involvement of RNA viruses, such as Rotavirus and Reovirus in the pathogenesis of BA (62).

Type 1 IFN signaling is part of a specific antiviral response resulting in enhanced major histocompatibility complex (MHC) expression so that infected cells can be recognized and killed easier by immune cells. Moreover, a differentiation of the immune response into a type 1 helper T-cell (Th1) specific response is promoted (12, 61). This is important for the adaptive immune response that follows the initial injury. Activation of TLR7 and type 1 IFN initiates production of interleukin-8 (IL-8) in BA by cholangiocytes and macrophages (58). IL-8 is a chemokine that attracts neutrophils mainly, but is also able to attract lymphocytes to the site of injury, such as natural killer cells (NK) which are part of the virus specific response as well (11, 63). NK cells are part of the innate immune response that can recognize and kill virus infected cells by recognition of the MHC complex (11, 63). Increased infiltration by NK cells in liver biopsies of BA patients compared to cholestatic controls has been reported (64, 65).

Tumor necrosis factor-related apoptosis inducing ligand (TRAIL) and CD95 (alternatively known as Fas) can be expressed by cholangiocytes. After administration of a double stranded RNA analog, TRAIL expression was found to be increased, in contrast to Fas, that remained unchanged. Signaling via type 1 IFN can lead to increased apoptosis by TRAIL and increased expression of these apoptotic receptors therefore suggests that activation of TLR3 leads to cholangiocyte apoptosis by expression of TRAIL (60).

**Figure 1:**
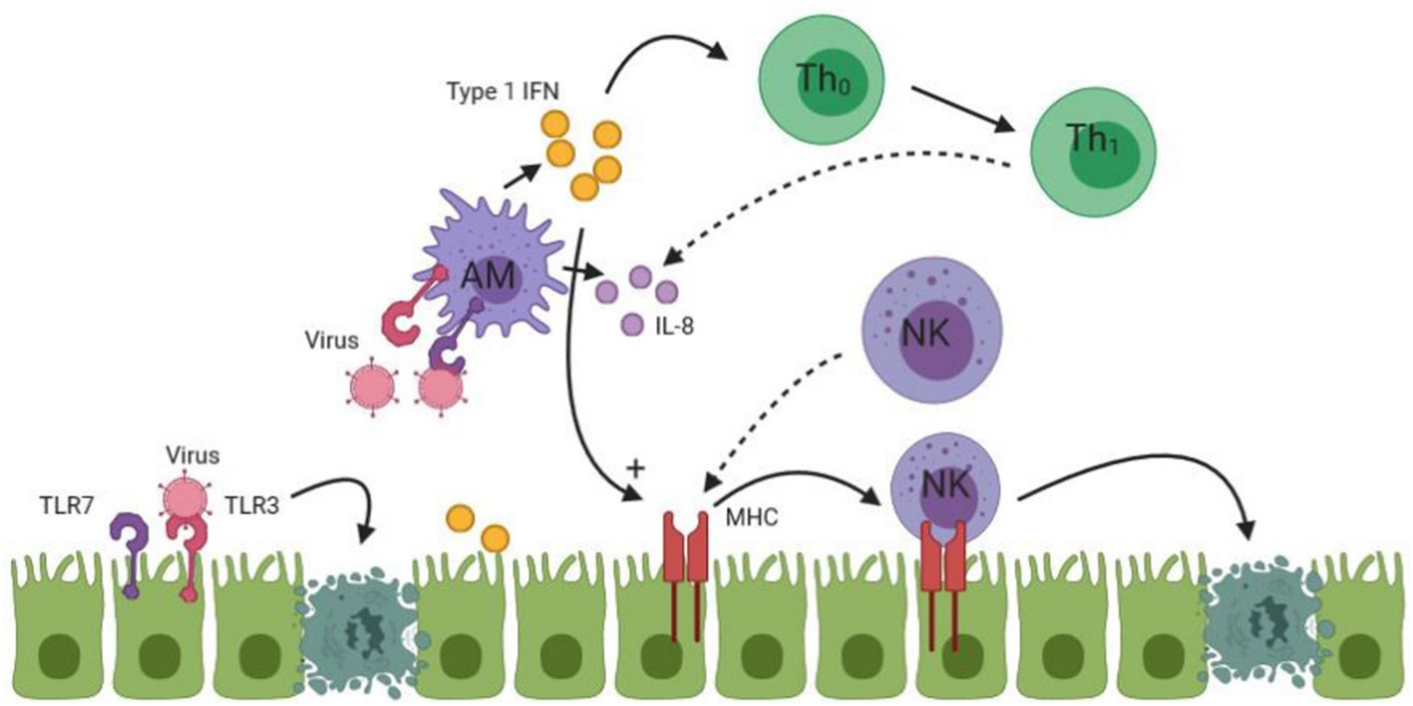
An overview of the initial immune response in BA. Undotted arrows indicate a consequence of activation. Dotted arrows in this figure indicate homing of lymphocytes to the site of injury. TLR, Toll like receptor; Th, T-Helper cell; AM, Activated macrophage; NK, Natural killer cell. See text for more details of the process.

In summary, the innate immune response in BA initiated by the activation of TLR3 and 7, which results in apoptosis of cholangiocytes by NK cells. Moreover, a viral specific immune response via type 1 interferons and other pro-inflammatory cytokines from cholangiocytes, macrophages and dendritic cells is initiated. An overview of the initial immune response is given in Figure 1.

### Adaptive Immune Response

IL-8 attracts effector immune cells to the site of injury as a result of TLR activation. When effector immune cells reach the biliary epithelium, the release of cytokines leads to proliferation and increased production of these effector immune cells and to a more tissue-specific immune response targeted at the cholangiocytes. The innate immune response stimulates and develops a more specific and potent adaptive immune response (12).

Effector cells of the adaptive immune response consist of CD4+ and CD8+ T-cells and phagocytes such as macrophages, among others (12). Macrophages play a special role in the immune system and in the pathophysiology of BA. Macrophages can recognize foreign material in the body but also have a function as effector cells of the innate and adaptive immune system. Macrophages fulfill this role by phagocytosis, a process where infected cells or cell debris is ingested by the macrophage and destroyed (11, 12). After activation, macrophages secrete tumor necrosis factor-alpha (TNF-α) and express CD68 (66). TNF-α is mainly produced by macrophages but can also be produced by other cells, such as cholangiocytes and T-cells (11, 12, 67). TNF-α is an important pro-inflammatory cytokine that can activate other immune cells but also lead to apoptosis of cells directly (12).

CD4+ T-cells are T-helper cells, which recruit phagocytes to mediate the destruction of target tissue. A subset of CD8+ T-cells are the cytotoxic T-lymphocytes (CTL). CTLs are able to kill cells that are infected with a virus. The target cell lacks the ability to eradicate the microbe that has infected the cell, so the only way to eradicate the virus is to destroy the entire cell; an action that is performed by the CD8+ CTL (or NK cell during innate immune response) (12). The effector T-cells have to be activated by a specific antigen/major histocompatibility complex (MHC) combination on the T-cell receptor (TCR). The interaction between a combination of antigen and MHC leads to the development of a very specific adaptive immune response (68). Proof of specific activation of T-lymphocytes was found by Mack et al. (68) who found expansion of CD4+ and CD8+ T-cells in BA tissue via a limited repertoire of T-cell receptors compared to controls. This indicates activation via a specific antigen.

Urushihara et al. found that in the liver specimens of patients with BA, there was an increased number and increased size of Kupffer cells at KPE and at LTx (69). This finding was replicated by Mack et al. (70). Moreover, IL-18, which is produced by these Kupffer cells, was also found to be significantly elevated in BA patients compared to controls. IL-18 is most potent when working together with IL-12 in the immune response. IL-12 is a differentiation factor that is released from macrophages to differentiate CD4+ T-helper cells into T-helper type 1 (Th1) cells (11, 12). In concert with IL-12, IL-18 induces the production of IFN-γ from Th1 cells (71). IFN-γ leads to further differentiation of the Th1 response and inhibition of the development of other T-cell lineages (12). Moreover, it activates macrophages to perform phagocytosis on infected cells and microbes (12). Mack et al. confirmed the presence of a Th1 immune response by finding extensive infiltration of CD4+ and CD8+ effector T-cells which produced IL-2, IFN-γ, and TNF-α (70). Furthermore, chemokine receptor CXCR3 is a homing factor specific for a Th1 immune response (11). CXCR3 was found to be up-regulated within portal tracts and biliary remnants in the livers of BA patients compared to controls (72). Whitington et al. investigated the presence of osteopontin (OPN) in the liver of BA patients (73). OPN is secreted by cholangiocytes and is responsible for the homing of macrophages to the bile ducts and is considered to further enhance Th1 differentiation (74). It appears that in BA, the innate immune response develops into a Th1 differentiated immune response which targets the cholangiocytes near the portal area.

**Figure 2:**
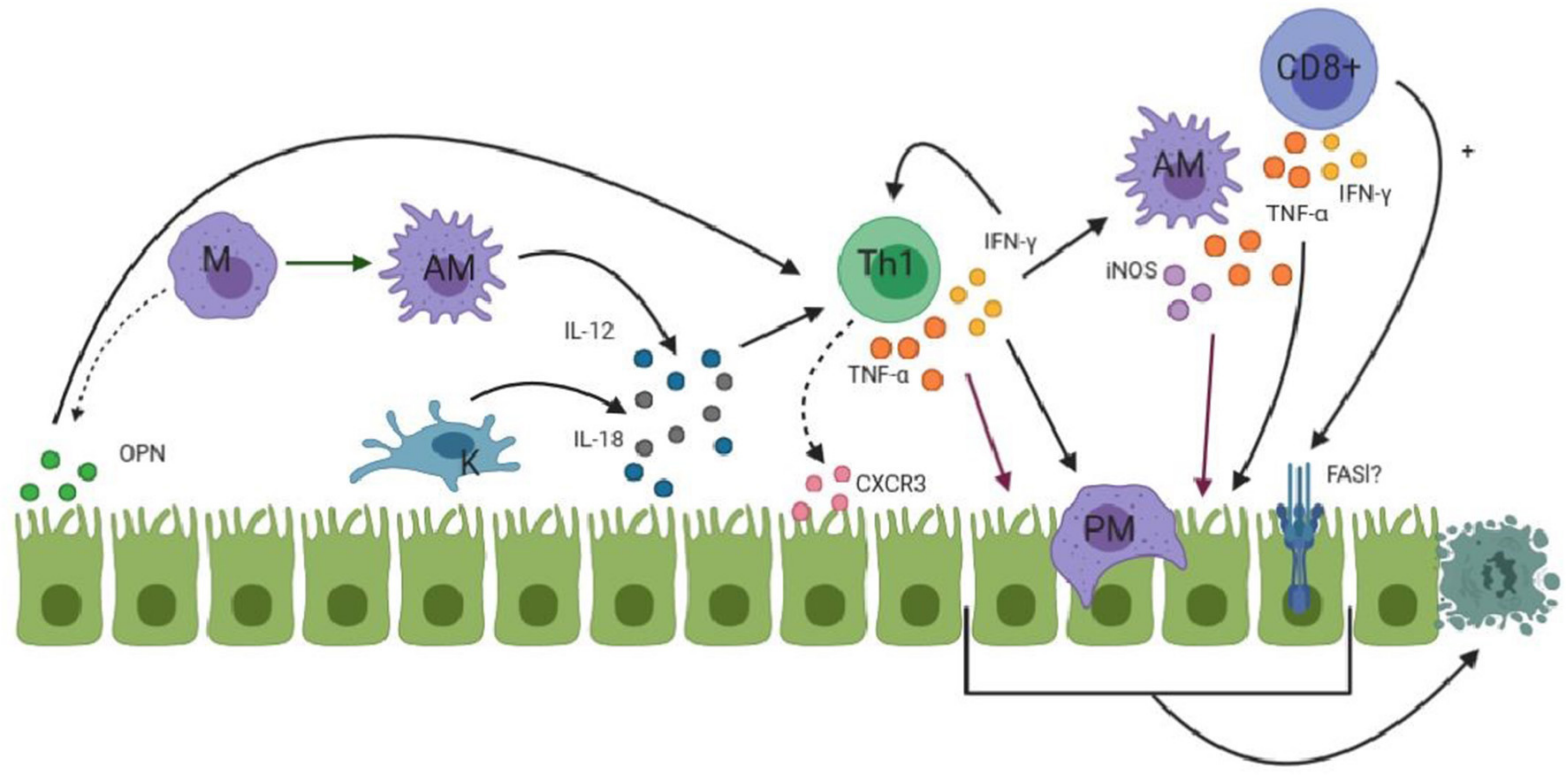
An overview of the process where macrophages (M, purple) are attracted to cholangiocytes (green) and the Th1 (green) response starts to develop. Activated macrophages (AM) are shown as spikey purple while Kupffer cells (K) are shown in blue. PM represents phagocytosing macrophage. FASl is shown with a question mark due to the fact that its involvement in apoptosis is not proven. Dotted arrows indicates that attraction of immune cells takes place. (OPN and CXCR3). More detailed information is given in the text.

During the innate immune response, NK cells are thought to kill cholangiocytes. Moreover, TLR3 activation results in their apoptosis. Macrophages can also kill cells by the production of reactive oxygen species (ROS), nitric oxide (NO) and lysosomal enzymes (11, 12). The role CD8+ CTLs play in the apoptosis of cholangiocytes remains to be determined. Ahmed et al. found a high expression of CTLs in BA patients, but not of the cytotoxic products of these CTLs (granzyme, perforin, and Fas ligand) (75). In contrast, Guo et al. found up-regulation of CD8+ CTLs and its co-stimulatory molecules in BA patients, indicating a toxic function exerted by these CTLs (65). Besides their cytotoxic function, CTLs are able to produce cytokines such as IFN-γ, thereby contributing to the Th1 immune response in BA (11, 12). In a murine model of BA, TNF-α, and IFN-γ were both required for the apoptosis of cholangiocytes (76). IL-8 is released from macrophages as a downstream product of type 1 IFN activation. IL-8 attracts neutrophils to the site of injury. Neutrophils have been studied in BA, but are thought to infiltrate the liver as a consequence of biliary tract obstruction, rather than playing a role in the initiation of bile duct damage. They may, however, play a role in cholangiocyte damage later on in the pathogenesis (77). An overview of the adaptive immune response in BA is shown in Figure 2.

## Progressive Liver Fibrosis in Biliary Atresia

Besides obliteration of cholangiocytes, progressive fibrosis of the liver is observed in the pathogenesis of BA; in particular in later stages of the disease. Continuing the hypothesis that an immune response is responsible for cholangiocyte damage and apoptosis, it seems likely that a continuation of the immune response plays a role in the progression of liver fibrosis. Most BA patients eventually require an LTx even if KPE is deemed successful (i.e., clearance of hyperbilirubinemia within 6 months after KPE), suggesting that other factors than cholestasis contribute to liver fibrosis as well in BA (3).

### Characteristics of Liver Fibrosis

Liver fibrosis can result from a variety of etiologies, such as infections, alcohol, and autoimmune disorders (78). Cholangiopathies lead to a distinct type of liver fibrosis which is termed biliary fibrosis (18). Liver fibrosis develops due to the transformation of hepatic stellate cells (HSC) into myofibroblasts (MF) and the excess deposition of components of the extracellular matrix (ECM) by MFs. Other cells can also contribute to liver fibrosis by transforming into MFs, such as portal fibroblasts (PF) (79). MFs start to produce collagen-1 and express α-smooth muscle actin (α-SMA) during liver fibrosis (79, 80). PFs and HSCs transform to their pro-fibrotic phenotype as response to pro-fibrotic growth factors such as transforming growth factor (TGF)-β and platelet-derived growth factor (PDGF). The pro-fibrotic growth factors can be produced by a variety of cells such as macrophages in the liver but also by MFs themselves, creating a vicious cycle (12, 81, 82). Macrophages mediate survival of MFs by secreting TNF-α so that MFs can maintain a pro-fibrotic environment (18). Different etiologies lead to a different balance of cell types that are being activated and lead to fibrosis, however HSCs remain the most important producer of pro-fibrotic MFs in most models of fibrosis and in cholangiopathies (83). In the current view, PFs are the initial responders to damage to cholangiocytes and they subsequently recruit HSCs as the main contributor to biliary fibrosis.

As response to injury, the liver is able to regenerate. Regeneration of the liver during injury in a cholangiopathy is mediated by replication and proliferation of already existing cholangiocytes and by the activation of hepatic progenitor cells (HPCs) that can both differentiate toward reactive ductular cells (RDCs) (18). Proliferation of cholangiocytes and HPCs into RDCs together with the niche of inflammatory cells surrounding this proliferation is termed the ductular reaction. The ductular reaction is a hallmark of pathological repair of the damage that is being (or has been) done to the cholangiocytes or hepatocytes (18). Initiation of the ductular reaction requires (re)activation of pathways that result in the proliferation of HPCs into biliary epithelial cells. These pathways are, among others the Notch and Wnt/β-catenin signaling pathways (18). Fabris et al. investigated the ductular reaction in Alagille syndrome (AGS) and BA. AGS is characterized by defective Notch signaling (84). The authors concluded that Notch is required for the differentiation of HPCs into RDCs, as AGS patients lacked RDCs in their livers. Notch signaling increased as liver fibrosis in BA progressed (84, 85). Wnt/β-catenin signaling is involved in the determination of cell fate and proliferation (86). β-catenin acts as a signal transducer of activated Wnt. In normal circumstances, β-catenin is removed from the cytoplasm of cells, thereby preventing activation of Wnt target genes (86). When the signaling pathway is activated, β-catenin is stabilized within the cell and allows transcription of target genes in the nucleus of the cell (86). In liver fibrosis, Wnt/β-catenin signaling enhances HSC activation and survival (87). Significant up-regulation of signaling proteins of the Wnt/β-catenin in the liver of BA patients compared to controls was observed. Furthermore, protein expression of products of the Wnt/β-catenin pathway correlated to hepatic fibrosis in BA, indicating involvement of this mechanism in fibrosis progression in BA (88). Recently, a downstream product of the Wnt/β-catenin pathway, a G-protein termed RhoU, was investigated in the biliatresone induced mice model (50). It was found that RhoU was upregulated in mice treated with biliatresone, indicating activation of the Wnt/β-catenin pathway. Subsequently, they analyzed the expression in livers of BA patients and found overexpression of RhoU compared to controls, supporting their model and indicating a potential role for the Wnt/β-catenin pathway in BA (50).

Notch and Wnt/β-catenin signaling pathways have been linked to epithelial-to-mesenchymal transition (EMT). During EMT, epithelial cells lose characteristics of epithelial cells, such as expression of E-cadherin and differentiate or transform into mesenchymal cells. Due to the loss of tight junction proteins, mesenchymal cells are able to migrate to other organs (89). EMT has been implicated in cancer metastasis and fibrosis (90). The existence and relevance of EMT remains controversial, however. Studies have investigated EMT in relation to BA and liver fibrosis, although the existence and relevance in BA remains undetermined (91). Although the Notch and Wnt β-catenin signaling pathways may not be involved by EMT in BA, they can still play a role by enhancing the proliferation of HPCs to RDCs or by activating HSCs.

Santos et al. investigated the hepatic expression of cytokeratin 7 (CK7), a marker of proliferation of cholangiocytes (i.e., ductular reaction), in patients with BA (92). They found that high CK7 expression in the liver at time of KPE predicted a poorer survival. Higher presence of ductular reactions may therefore be an indication of the presence and extent of liver fibrosis. Kerola et al. (93) found increased hepatic gene expression of α-SMA in BA patients despite receiving a successful KPE, indicating the presence of activated MFs. The hepatic gene expression of α-SMA correlated with the extent of fibrosis, expansion of the ductular reaction and was located around ductular proliferations of cholangiocytes. The cohort of Kerola et al. only contained successfully treated BA patients, suggesting other factors than cholestasis can also play a role in the progression of the ductular reaction and subsequent liver fibrosis.

Angiogenesis, the formation of new blood vessels, is a process that occurs during the growth and repair of organs. The process of angiogenesis is influenced by growth factors and hypoxia (i.e., a shortage of oxygen) (94). Angiogenesis consists of multiple sequential phases and can be induced by inflammation or hypoxia. The most important mediator during all steps of angiogenesis is members of the vascular endothelial growth factor (VEGF) family, with VEGF-A as the main component (94). Hepatic fibrosis is an inflammatory process with abnormal wound healing, which can influence angiogenesis by formation of pro-angiogenic cytokines and induction of hypoxia (95). Hypoxia can occur due to the anatomical changes during hepatic fibrosis; reduced fenestration of sinusoidal cells and accumulation of fibrotic tissue makes it more difficult for oxygen to be transported to the cells (96). During inflammation, Kupffer cells can induce the release of angiogenic factors such as VEGF by the production of oxygen radicals, such as ROS (96).

Angiogenesis in relation to BA is less well-studied and the results of the studies performed varied. In these studies, the expression of members of the VEGF family in biliary ducts and arterial walls in liver biopsies. Allam et al. found that a significant higher proportion of BA patients had positive VEGF-A (i.e., most prominent of VEGF family) staining compared to cholestatic controls (97). Moreover, they found that significantly more BA patients had positive VEGF-A expression when they received an unsuccessful KPE compared to BA patients receiving a successful KPE. Edom et al. found a characteristic staining of VEGF-A in liver biopsies of BA patients (98). They propose that there is a higher expression of VEGF-A due to hypoxia induced by portal expansion of the biliary tract. Alternatively, the increase in VEGF-A expression may be due to the fact that higher blood supply is required for the ductular reaction and proliferation of cholangiocytes. Another study found that there was increased gene expression of hypoxia-inducible factor (HIF)1a and HIF2a (99). Usually, HIF expression leads to induction of VEGF-A (100). Surprisingly, Fratta et al. found that BA patients with a high expression of HIF1a and HIF2a had a significantly lower expression of VEGF-A and its related receptor, VEGFR2, than patients with a low expression of HIF1a and HIF2a. Moreover, VEGF-A negatively correlated to expression of both HIF1a and HIF2a (99). The authors explained their findings by hepatobiliary ischemia due to an insufficient angiogenic response to the liver hypoxia. Alternatively, decreased expression of VEGF-A and VEGFR2 may be due to reduction of blood supply by blockage of the bile duct. This is contrasting with previous findings, where VEGF-A expression was correlated with cholangiocyte proliferation, a feature associated with increased liver fibrosis. Interestingly, genistein, an isoflavonoid, suppresses VEGF-A and VEGF receptor expression. Genistein can be transformed into biliatresone in the human intestine by bacteria (101).

In summary, there appears to be proliferation of cholangiocytes in patients with BA. Pathways such as the WNT/β-catenin pathway may be involved in this process. Angiogenesis is a process that is related to liver fibrosis and cirrhosis. In BA, angiogenesis has been investigated and the results have been variable. Increased or decreased VEGF-A expression may be related to biliary proliferation or an insufficient angiogenic response, respectively. More studies are required to explore the relation between cholangiocyte proliferation and induction of angiogenesis by VEGF-A in BA. Moreover, the impact biliatresone has on VEGF-A expression in the BA model could be further explored.

### Immune Response and Liver Fibrosis in Biliary Atresia

Kobayashi et al. (102) found increased numbers of activated macrophages (expressing CD68) in liver biopsies of BA patients compared to controls with neonatal hepatitis. At time of KPE, patients that received an unsuccessful KPE had higher number of activated macrophages compared to BA patients that received a successful KPE. This indicates that already at KPE, the extent of macrophage proliferation and immune response can determine the outcome of BA patients. Interestingly, another study found that even despite clearance of jaundice in BA patients, serum IL-18 and the number of Kupffer cells in the liver remained elevated after KPE (69). Furthermore, Narayanaswamy et al. (103) investigated the presence of various interleukins and pro-inflammatory cytokines in the serum of BA patients at multiple time points. They observed that 6 months after KPE, BA patients had significantly higher serum values of IL-2, IFN-γ, IL-4, IL-18, and TNF-α when compared to cholestatic controls. Most cytokines appeared to increase in concentration during this 6 month period. Although there was no difference in these cytokines in the serum of BA patients who underwent a successful KPE and patients in whom the KPE was unsuccessful, the authors found significant differences when they divided the group of BA patients in those who underwent an LTx and those who still survived with their native liver; all cytokines except IL-18 were significantly elevated at 6 months post-KPE in patients requiring early LTx.

There appears to be a continuing immune response that may be responsible for the faster liver fibrosis and subsequent LTx. Moreover, the fact that patients who are able to clear their hyperbilirubinemia and patients that are not able to clear it do not have a significant different cytokine profile over 6 months indicates that cholestasis is not the sole mechanism responsible for the progressive liver fibrosis. Macrophages may play a role in the continuation of the immune response, contributing to hepatic fibrosis by inhibiting apoptosis of MFs by secreting TNF-α, for example.

In summary, there is an initial immune response responsible for cholangiocyte damage and apoptosis. This immune response is continued into a pro-fibrotic reaction that eventually leads to liver fibrosis, despite clearance of cholestasis. Cholangiopathies can lead to liver fibrosis via cholestasis and a ductular reaction that takes place as a response to injury. The ductular reaction is defined as proliferating cholangiocytes with associated pro-inflammatory proteins (18). It is likely to think that pathways such as Wnt/β-catenin and a continuation of the inflammatory response together lead to progressive liver fibrosis in BA. The maintenance of the pro-fibrotic environment can for example be caused by the vicious cycle HSCs create by producing their own pro-fibrotic growth factors (82), but also by a yet unidentified factor such as a matrix metalloprotease (MMP).

## Matrix Metalloproteinases: Functions and Role in Pathology

Matrix metalloproteinases (MMPs) are a big family of proteases that use zinc to mediate their proteolytic activity (22, 24). Their first function to be described was the turnover and modulation of the ECM by proteolytically degrading various proteins that are present in the ECM. The ECM is a mixture of cells and non-cellular components which function as a physical scaffold to many cells. The two classes of molecules that mainly constitute the ECM are proteoglycan (PG) and fibrous proteins, such as collagen and elastin (104). PG forms a hydrophilic gel that has many differing functions such as interacting with growth factors, cell receptors and cytokines, while collagen gives the cell tensile strength (104, 105). This allows the ECM to send signals to the cell that regulate different cell functions, such as differentiation and adhesion (105). By altering the structure and components of the ECM, there is an alteration of the specific signals that are transmitted to and from the cell, affecting its behavior. In order to maintain homeostasis between the ECM and the cell, it is important that the matrix is remodeled (104, 105). Disbalance of homeostasis in the ECM leads to pathological conditions. Too little remodeling leads to accumulation of collagens and subsequent fibrosis. Too much turnover of ECM can lead to collagen loss resulting in cardiomyopathy due to decreased contractility, for example (106).

MMPs are the most important mediators of remodeling of the ECM. As a collective group, the MMPs are able to cleave all the components of the ECM (106). There are more than 20 different MMPs and they are subdivided into groups based on their substrate and their structure (gelatinases, collagenases) (22, 24). All MMPS have in common that they contain a catalytic domain with a zinc ion. When MMPs are in their inactive form (zymogen), the pro-domain containing cysteine binds to the catalytic Zn^2+^ ion in the catalytic domain of the MMP so that catalysis is prevented. This structure is the so called “cysteine switch” (22, 107). When the MMP is activated the pro-domain is cleaved off, thereby freeing the Zn^2+^ ion and allowing catalytic activity. Cleaving of the pro-domain of MMPs can be done by various mechanisms, such as cleavage by plasmin, proprotein convertases, but also by other MMPs (107).

Besides ECM turnover, MMPs play a role in inflammation, vascularization, cell migration and proliferation, among others (22). Each MMP has unique non-ECM related functions which results from the activation of bioactive proteins, a process known as ‘shedding’. In inflammation, for example, MMPs can regulate the movement of leukocytes into the tissue by creating a chemokine gradient (30). For example, in a mouse model of allergic inflammation in the airway there was a reduction of the number of eosinophils in the bronchoalveolar lavage in mice with knockout MMP-9. The authors show that there was disruption of chemokine gradients due to the absence of MMP-9 (108). As mentioned briefly in the introduction, MMPs also play a role in cancer by enhancing angiogenesis. For example, MMPs promote angiogenesis by degrading ECM components so that endothelial cells can migrate to the location where new vessels are being formed (109). MMP-14 is one of the MMPs that fulfills this role. Moreover, MMP-14 is required for the production of vascular endothelial growth factor (VEGF), directly promoting angiogenesis (110).

MMPs are linked to various pathological processes and diseases. Most obvious, they play a role in fibrosis of the liver and the lung and other conditions in which alteration of the ECM leads to pathology, such as aortic aneurysms (24). They can have a pro- or anti-fibrotic role depending on where in the process of fibrosis they exert their proteolytic effect. Moreover, they can also indirectly affect fibrosis by the alteration of regulatory factors. In physiologic conditions, MMPs are secreted by a variety of cell types, such as by epithelial cells or inflammatory cells such as macrophages, when they are needed (22). Their activity is regulated by tissue inhibitors of metalloproteinases (TIMP). Pathologic conditions arise due to excess activity of MMPs or reduced inhibition of MMPs by TIMPs (22).

In hepatic fibrosis for example, MMP-9 exerts a pro-fibrotic effect by mediating leukocyte trafficking while it also correlates to the extent of hepatic inflammation but not fibrosis (111). Moreover, HSCs are thought to be activated by MMP-2 and 14 while MMP-13 leads to shedding of TGF-β (112, 113). Surprisingly, MMP-2 and 14 are also linked to fibrolysis due to their collagenolytic activity. MMP-2 and 14 activity remained elevated after termination of the administration of the toxic CCL_4_ in an animal model of liver fibrosis, suggesting a role in the resolution of fibrosis. Resolution of fibrosis occurred in concert with a decrease of TIMP activity, indicating an imbalance leading to increased activity of MMP-2 and 14 (114). This further indicates that the role of MMPs in liver fibrosis can vary depending on the substrate and the timing in the process of liver fibrosis. It requires further investigation to clarify the sources of production of MMPs and their effects at different stages of fibrosis.

## Matrix Metalloproteinase-7 (MMP-7)

MMP-7 or Matrilysin-1 belongs to the group of matrilysins. MMP-7 can only degrade collagen type IV and is not able to cleave other collagens, which are the major constituents of the ECM (105). However, MMP-7 is able to degrade fibronectin, gelatin type I, III, IV and V (degraded collagen), laminin, entactin (alternatively known as nidogen) and elastin (115). In the liver, MMP-7 is thought to be produced by a variety of cells, such as glandular epithelial cells, cholangiocytes but also by macrophages (33, 116, 117).

In the ECM of the liver, collagen type IV, laminin and entactin form the main constituent of the basement membrane, which forms the outer barrier of epithelial cells (118). Fibronectin, on the other hand, is a component of the ECM that is responsible for cell to cell adhesion by binding to integrin (119). In hepatic fibrosis, the deposition of basement membrane proteins such as collagen IV actually increases (118). This could explain why MMP-7 has been described more in cancer, where disruption of the basement membrane leads to the ability of cancer cells to invade intact tissue (120). In liver fibrosis, the role of MMP-7 has not been described extensively. A recent trial, however, found that serum MMP-7 was a reliable biomarker of advanced liver fibrosis and cirrhosis (121).

As mentioned previously, VEGF is one of the most important inducers of angiogenesis (94). It is thought that VEGF is expressed in a quiescent state in normal tissue and remained in a non-active state by binding to VEGF inhibitors, such as soluble VEGF receptor-1 (sVEGFR-1). In this manner, endothelial cells are protected of angiogenesis by the inhibition of VEGF (122). Although this mechanism is not yet proven in many different cell types, a study has shown that MMP-7 is responsible for the degradation of sVEGFR-1. This will lead to liberation and activation of VEGF and subsequent initiation of angiogenesis (123). MMP-7 thus appears to exert a pro-angiogenic function by affecting the balance between VEGF and sVEGFR-1.

MMP-7 has been investigated in BA and BA-related liver fibrosis. MMP-7 has been identified as a reliable biomarker for the diagnosis of BA in cholestatic patient cohorts by different research groups (19–21, 33, 124, 125). Lertudomphonwanit et al. found that MMP-7 was released by cholangiocytes in response to injury, although it was found in an experimental model of BA in mice (125). Interestingly, using RNA sequencing techniques, it was found that there was upregulation of the MMP-7 gene in liver samples of mice with BA induced by biliatresone (48).

Bezerra et al. found that MMP-7 was one of the genes that is up-regulated in the livers of BA patients compared to cholestatic controls (126). Hsieh et al. found that there was a progressive increase of MMP-7 gene expression from KPE to LTx in BA patients (127).

Hepatic expression of MMP-7 correlated with the extent of liver fibrosis in two cholestatic patient cohorts (20, 21). In a cohort of successfully treated BA patients, hepatic expression of MMP-7 was localized in biliary epithelium where ductular proliferation took place (32). MMP-7 co-localized with CK7, which is a marker of cholangiocyte proliferation and the ductular reaction. In contrast to previous studies, hepatic expression of MMP-7 correlated to the Metavir liver fibrosis stage despite a successful KPE indicating that hepatic overexpression of MMP-7 can occur independent of cholestasis. Huang et al. found that MMP-7 was expressed by more cell types, such as Kupffer cells, as the fibrosis progressed in BA patients (33). Jiang et al. found a correlation between serum MMP-7 concentration, age and fibrosis stage in BA patients, suggesting that serum MMP-7 plays a role in fibrosis starting from a very young age in BA patients (21). Despite these correlations of MMP-7 with extent of liver fibrosis, a clear demonstration of the longitudinal effects that MMP-7 has on liver fibrosis in BA is lacking.

These results provide circumstantial evidence that MMP-7 is involved in the pathophysiology of BA. MMP-7 can be involved during the initiation of cholangiocyte damage leading to BA as well as during the progression of liver fibrosis that is observed in these patients. However, the mechanism by which MMP-7 accomplishes either or both of these processes remains to be elucidated. In liver fibrosis, the role of MMP-7 has not been accurately described. MMP-7 has been more accurately described in other pathologies, such as pulmonary and renal fibrosis, which can be used to generate hypotheses about the involvement of MMP-7 in BA.

## MMP-7 IN Billiary Atresia: Lessons Learned From Other Pathologies

### Renal Fibrosis

Renal fibrosis is often caused by the overproduction of ECM by interstitial fibroblasts (128). The exact mechanism of renal fibrosis remains elusive. Although controversial, one of the proposed mechanisms of renal fibrosis is EMT. MMP-7 has been investigated in renal fibrosis in light of EMT and thought to be involved by a variety of mechanisms (129). In kidney fibrosis the WNT/β-catenin signaling pathway is activated when there is injury of tubular epithelial cells, leading to the transcription of target genes by β-catenin. In cancer and renal fibrosis, MMP-7 is one of the downstream molecules transcribed by β-catenin (130). MMP-7 subsequently induces apoptosis of epithelial cells by activating Fas ligand and degrading collagen IV and laminin, which destroys the basement membrane of renal tubular epithelial cells, supposedly leading to EMT and subsequent kidney fibrosis. Although EMT may not be the mechanism by which renal fibrosis occurs, MMP-7 is thought to be actively involved in the mechanism of renal fibrosis because the extent of renal fibrosis was markedly reduced in MMP-7 gene knockout mice compared with wildtype mice (130). Furthermore, pharmacological inhibition of MMP-7 reduced the extent of renal fibrosis markers (130).

**Figure 3:**
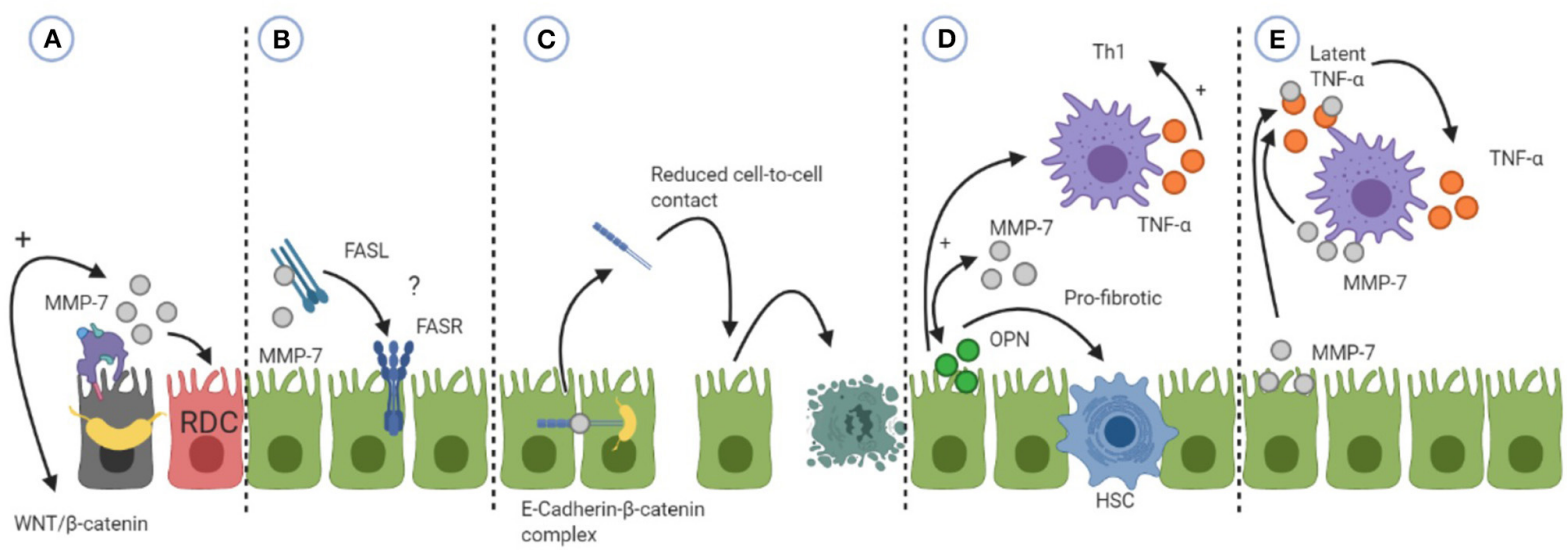
An overview of how MMP-7 can be involved in the pathophysiology of biliary atresia (BA). **(A)** MMP-7 can be a result of activated WNT/β-catenin signaling leading to proliferation of cholangiocytes into reactive ductular cells (RDC). **(B)** MMP-7 can activate Fas-ligand (FASL) which can subsequently induce apoptosis by binding to the Fas-receptor (FASR). The relevance of this mechanism is unsure, hence the question mark. **(C)** MMP-7 can shed E-cadherin leading to reduced cell-to-cell contact and subsequent apoptosis. **(D)** MMP-7 and osteopontin (OPN) can engage in a positive feedback loop. OPN can exert its pro-fibrotic effects by activating hepatic stellate cells (HSC) or its Th1 differentiating effects by activating macrophages (M). **(E)** MMP-7 can activate tumor necrosis factor alpha (TNF-α) that is secreted by macrophages, enhancing its pro-inflammatory effects. See the text for more details of the mechanism.

In BA, EMT is also controversial. Wnt/β-catenin signaling has been shown to be activated during liver fibrosis in BA (88). As Wnt/β-catenin is involved in the regulation of cell fate, the signaling pathway is thought to enhance the ductular reaction that is seen in liver fibrosis in BA. The increased hepatic expression of MMP-7 in BA may therefore be a result of activated Wnt/β-catenin signaling, which leads to hepatic fibrosis via initiation of the ductular reaction (Figure 3A). Moreover, Fas is thought to be expressed on cholangiocytes in BA, leading to apoptosis. Enhanced MMP-7 expression can lead to activation of FasL resulting in apoptosis (Figure 3B). Although varying results concerning Fas and BA have been found, the relation between cholangiocyte apoptosis, Fas and MMP-7 should be further explored.

E-cadherin is a transmembrane protein present at cell junctions, providing cell-to-cell integrity (119). In kidney fibrosis, MMP-7 can disrupt cell-to-cell integrity by shedding of E-cadherin. E-cadherin is characteristic of epithelial cells and loss of E-cadherin expression is considered one of the first steps in EMT. In BA, the hepatic expression of E-cadherin was investigated. Hepatic expression of E-cadherin in BA patients was reduced compared to cholestatic controls (131, 132). Moreover, there was an inverse correlation between hepatic E-cadherin and apoptosis of cholangiocytes in BA patients, suggesting that reduced expression of E-cadherin can lead to apoptosis of cholangiocytes (131). Harada et al. found reduced expression of E-cadherin in damaged cholangiocytes in BA patients compared to non-damaged cholangiocytes (132). As MMP-7 is able to shed E-cadherin, it would be of great interest to investigate if MMP-7 can lead to cholangiocyte apoptosis via reduction of cell-to-cell adhesion by shedding of E-cadherin (Figure 3C).

β-catenin is attached to the intracellular domain of E-cadherin (133). In theory, when E-cadherin is shedded, β-catenin is freed and able to translocate to the nucleus of the cholangiocyte. Then, target genes of the Wnt/β-catenin can be transcribed, of which MMP-7 is one (130). Via this mechanism, a potential positive feedback loop between β-catenin and MMP-7 would be possible. However, no difference in the intracellular expression of β-catenin between control and BA livers and no nuclear accumulation of β-catenin were found in cholangiocytes in a study investigating this (131). Therefore, a positive feedback loop via this mechanism seems unlikely. However, as β-catenin may be involved in the pathogenesis of BA, it would still be of interest to further investigate the involvement of β-catenin and related signaling proteins in relation to shedding of E-cadherin specifically in BA (88).

### Pulmonary Fibrosis

The role of MMP-7 has also been thoroughly investigated in idiopathic pulmonary fibrosis (IPF), a rapid progressing fibrotic lung disease affecting the alveolar epithelial cells (AEC) (134). MMP-7 can be used as a biomarker that predicts mortality in patients with IPF (124). Moreover, Fujishima et al. showed that proMMP-7 is produced locally by hyperplastic AECs and macrophages in patients with IPF indicating an active role of MMP-7 in the fibrotic microenvironment (116). Similarly to renal fibrosis, MMP-7 is able to shed E-cadherin in alveolar epithelial cells, leading to activation of β-catenin signaling and by the induction of Fas, leading to apoptosis of AECs (135). However, this activation is considered as a physiological process to some extent in the repair of epithelium in the airways (136).

The pulmonary expression of MMP-7 has also been investigated in concert with osteopontin (OPN). Besides a Th1 cytokine, OPN is believed to play a role in fibrosis by recruiting and differentiating MFs (137, 138). Pardo et al. found that MMP-7 and OPN co-localize on alveolar epithelial cells in patients with IPF (139). Since MMP-7 is able to cleave, as well as to be cleaved by OPN, the authors propose that a positive feedback loop between MMP-7 and OPN is possible. This loop leads to a sustained activation and exertion of the effects of MMP-7 and OPN, causing rapid fibrosis in the lungs of patients with IPF (139, 140). OPN exerts its pro-fibrotic effect in the lungs by increasing the secretion of collagen-1 by fibroblasts while in AECs it induced the expression of MMP-7 (139). OPN has been investigated in liver pathologies (141). In liver fibrosis, OPN mediates its pro-fibrotic effects through the activation of hepatic stellate cells (HSC) to up-regulate the deposition of collagen-1, one of the hallmarks of liver fibrosis (79). OPN supposedly accomplishes this by two pathways, either by signaling to NF-kB in HSCs or by signaling biliary epithelial cells (BEC) to up-regulate TGF-β. Moreover, in cardiac MFs, it was shown that OPN is required for TGF-β induced differentiation of MFs, indicating that OPN is important for the induction of fibrosis (138). In BA, Whitington et al. showed that OPN can be secreted by BECs and is greatly overexpressed in areas of proliferating ductal cells (73). Moreover, OPN expression in the livers of BA patients correlated very strongly to the degree of hepatic fibrosis, but also to the gene expression of TGF-β (142). It is tempting to speculate that OPN and MMP-7 may be engaged in a positive feedback loop in BA, leading to the progressive liver fibrosis that is observed in a similar manner as in IPF (Figure 3D). Future research should explore the mechanism by which MMP-7 and OPN interact with each other in BA. It would for example be interesting to investigate which protein induces the expression of the other and what the sources of these proteins are.

Moreover, OPN is also considered a Th1 cytokine (74). A positive feedback loop between MMP-7 and OPN can also result in a progressive inflammatory response and subsequent hepatic overexpression of both proteins in BA patients. MMP-7 can influence the inflammatory response indirectly by activating OPN. OPN is able to regulate the migration and differentiation of macrophages during the innate immune response. After OPN activated dendritic cells (DCs) and macrophages, these cells start to produce TNF-α and IL-12 so that naïve T-cells are polarized into IFN-γ producing Th1 cells (74) (Figure 3D).

### Inflammation and Angiogenesis

MMPs can play a role in inflammation by a variety of mechanisms, such as by creating chemotactic gradients leading to influx of inflammatory cells into site of injury or by activating cytokines (30). MMP-7 activates intestinal pro α-defensins in mice, enhancing mucosal immunity (143, 144). α-Defensins are produced by Paneth cells in the intestinal crypts and have a direct antimicrobial effect and amplify the immune response by acting as a chemoattractant for immune cells. MMP-7 can also be produced by Paneth cells (143). MMP-7 has recently shown to promote an increased intestinal permeability by activating α-Defensins in Paneth cells of mice (144). Increased intestinal permeability leads to the leakage of products via the bloodstream and translocation to the liver causing inflammation. This is termed the gut-liver axis. The gut-liver axis has also been investigated in BA (145). As MMP-7 has not yet been identified in human intestinal Paneth cells, we will not discuss MMP-7 and the gut-liver axis in relation to BA here. However, this may be an interesting direction for future research. MMP-7 in relation to macrophages has been investigated in the resorption of herniated discs, a major cause of lower back pain (146). Haro et al. used a mice model to show that macrophages are responsible for the production of MMP-7 and latent TNF-α. The function of MMP-7 here is to cleave latent TNF-α to its soluble, active form. Activated TNF-α mediates macrophage invasion of the discs which is required for absorption of the disc. Importantly, the authors also show that MMP-7 is able to cleave latent TNF-α in isolated macrophages, indicating that MMP-7 ability to activate TNF-α is not restricted to the environment of herniated discs. Macrophages and its secreted TNF-α mediate damage in BA. Moreover, Kupffer cell expression of MMP-7 increases as liver fibrosis in BA progresses. MMP-7 may fulfill its role in the pathophysiology of BA by enhancing the activation of TNF-α from Kupffer cells and macrophages in the liver (Figure 3E).

In the context of inflammation, we will also discuss the role MMP-7 may play during angiogenesis in BA. As previously illustrated, findings on VEGF-A expression are variable. VEGF-A can be related to cholangiocyte proliferation because their proliferation requires a higher blood supply (97, 98). Alternatively, a lower expression of VEGF-A when BA patients experienced intense hypoxia in their liver, can be explained by an insufficient angiogenic response (99). It has been shown that MMP-7 is able to liberate VEGF-A by degrading sVEGFR-1 (123). MMP-7 localizes at the biliary epithelium, where the ductular reaction took place (32). It may be that MMP-7 is involved in the facilitation of angiogenesis by liberating VEGF-A in BA. Although the mechanism of VEGF-A liberation by MMP-7 is first to be demonstrated in liver tissue before adequate research in BA can take place, it may be a target of future research. Moreover, the exact role of VEGF-A and angiogenesis in BA should be further explored, especially in relation to progression of liver fibrosis, cholangiocyte proliferation and potentially MMP-7 as well.

## Clinical Implications

Exploring and establishing the exact role that MMP-7 has in BA can provide new therapeutic targets that can be explored in order to prevent liver fibrosis or even prevent BA from occurring. Anti-inflammatory medication in the form of corticosteroids has been investigated on numerous occasions and shown to be somewhat effective in improving outcomes, such as clearance of jaundice, in BA patients (147). However, as BA is an immune mediated disease, one would expect anti-inflammatory medication to be more effective. Higher effectiveness of perioperative medication can be achieved by selective inhibition of a pathogenic factor. MMP-7 may be a key mediator linking inflammation and fibrosis that can be accurately blocked by targeted medication. In mice with renal fibrosis, MMP-7 was blocked by MMP inhibitor II. The mice had reduced extent of renal fibrosis and a reduction of renal expression of collagen-1 was observed (130). Given this potential, the effect blocking MMP-7 has on the development of BA should be further explored.

Moreover, as serum MMP-7 correlates to the hepatic expression of MMP-7, opportunities for prognosis estimation can be explored (19–21, 33, 93). Almost all patients with BA undergo a KPE and in half of these patients, an LTx is required within the first 2 years after KPE (13). A prognostic factor that gives an estimation of the damage that is already done to the liver can help surgeons in deciding whether it is even beneficial to perform a KPE or to perform a primary LTx. Moreover, a prognostic factor after performance of a KPE can also be beneficial in BA patients. As of yet there is no factor that can accurately predict the need for an LTx. In other pathologies, MMP-7 as serum or urine marker correlates very well to clinical symptoms as well as prognosis (124, 130, 148). By having an accurate postoperative prognostic factor in BA, better triage of follow-up care for BA patients can be performed. Besides, measuring serum MMP-7 is also a lot less invasive than current diagnostic options, such as liver biopsy or ERCP (1). Before serum MMP-7 can actually be used as a prognostic biomarker, studies should investigate how hepatic MMP-7 expression progresses in the same BA patients over time; from KPE to LTx, for example. By doing this, a more accurate description of the effects of MMP-7 in BA-related fibrosis can be made. Subsequently, the predictive power of serum MMP-7 for native liver survival and occurrence of LTx in BA patients should be explored. This should be investigated as a single factor as well as in addition to other predictive markers such as postoperative bilirubin levels.

## Discussion

The aim of this review was to describe if and how MMP-7 can play a role in the pathophysiology of BA. An overview was given of the pathophysiology of BA, which is characterized by a Th1 immune response. Macrophages play a central role in the initial damage to cholangiocytes as well as in the progression to liver fibrosis. We described the structure and functions of MMP-7 in a physiological and pathological environment. In BA, hepatic MMP-7 expression correlates with the extent of liver fibrosis, even with minimal cholestasis after the performance of a KPE. MMP-7 co-localized with CK-7, a marker of cholangiocyte proliferation, and correlated to the extent of cholangiocyte proliferation (32). Moreover, MMP-7 localized near Kupffer cells, the residing macrophages of the liver. The co-localization of MMP-7 and important mediators of hepatic injury and fibrosis provides circumstantial evidence that MMP-7 is actively involved in the initial cholangiocyte injury in BA as well as in BA-related liver fibrosis. We described how MMP-7 is involved in other pathologies such as renal and pulmonary fibrosis and if these mechanisms could be extrapolated to the pathogenesis of BA. We hypothesized that the Wnt/β-catenin signaling pathway may be activated by MMP-7 in BA. Moreover, shedding of E-cadherin by MMP-7 leading to reduced cell-to-cell integrity and subsequent cell damage may occur in BA. In pulmonary fibrosis, OPN and MMP-7 are hypothesized to engage in a positive feedback loop leading to progressive fibrosis (139). A similar mechanism of positive feedback could well be at play in the progression of liver fibrosis in BA. Angiogenesis is a feature of liver fibrosis and cirrhosis. In BA, more research is required to clarify the role and relevance of angiogenesis and VEGF-A in the pathogenesis of BA. Due to the potential mechanisms of MMP-7 involvement in BA described, we think that the role MMP-7 can play in BA is primarily aimed at facilitating inflammation and fibrosis.

By generating hypotheses, we aimed to clarify the potential role of MMP-7 and to provide new targets for future research in BA. Moreover, determining whether MMP-7 plays an active role in the pathogenesis of BA or if MMP-7 is a marker of abnormal tissue regeneration will also provide clarity for future research. Active involvement of MMP-7 in the pathogenesis in of BA would imply possibilities for new therapeutic targets as well as new possibilities to estimate the prognosis of BA patients.

Limiting factors to this review were the fact that BA remains a very rare disease. Consequently, the studies that have been used to create this review range in their year of publication by 30 years. Moreover, BA is a heterogeneous disease with subtypes. In this review, we have tried to describe features of the isolated form of BA, but this was not specified in every study that was used. Subsequently, multiple subtypes of BA could have been included in patient cohorts, leading to different outcomes than if isolated BA only was included. We attempted to only include studies that focus on studies that investigated BA in humans so studies performed in mice with experimental BA were excluded. Future research should investigate the pathogenesis of BA in a specified, global cohort of BA patients, so that research around the globe can build further in a reliable way on results that are found.

In conclusion, BA and MMP-7 have an interesting relationship which is yet to be specified. MMP-7 can be involved in the pathogenesis of BA by multiple mechanisms that enhance inflammation, fibrosis or both. Future research can elucidate the role of MMP-7 in these processes further, so that application of MMP-7 as therapeutic target and prognostic biomarker can be explored.

## Author Contributions

MN: study concept and drafting of the manuscript. LB, JH, and PO: study concept and critical revision of the manuscript for important intellectual concept. HV: critical revision of the manuscript for important intellectual concept. All authors contributed to the article and approved the submitted version.

## Conflict of Interest

The authors declare that the research was conducted in the absence of any commercial or financial relationships that could be construed as a potential conflict of interest.

## Abbreviations

α-SMA: alpha-smooth muscle actin
AEC: alveolar epithelial cell
BA: biliary atresia
CC: choledochal cysts
CD: cluster of differentiation
CK7: cytokeratin 7
CTL: cytotoxic T-lymphocytes
DC: dendritic cell
ERCP: endoscopic retrograde cholangio-pancreatography
EMT: epithelial-to-mesenchymal transition
ECM: extracellular matrix
HPC: hepatic progenitor cell
HSC: hepatic stellate cell
IPF: idiopathic pulmonary fibrosis
IFN: interferon
IL: interleukin
KPE: Kasai portoenterostomy
LTx: liver transplantation
MHC: major histocompatibility complex
MF: myofibroblast
NK: natural killer
NLS: native liver survival
OPN: osteopontin
PAMP: pathogen associated molecular pattern
PRR: pathogen recognizing receptor
PF: portal fibroblasts
RDC: reactive ductular cells
Th1: T-helper 1 cell
TLR: toll-like receptor
TGF-β: transforming growth factor-beta
TNF-α: tumor necrosis factor alpha
VEGF: vascular endothelial growth factor.
